# Characteristics of Networks of Interventions: A Description of a Database of 186 Published Networks

**DOI:** 10.1371/journal.pone.0086754

**Published:** 2014-01-22

**Authors:** Adriani Nikolakopoulou, Anna Chaimani, Areti Angeliki Veroniki, Haris S. Vasiliadis, Christopher H. Schmid, Georgia Salanti

**Affiliations:** 1 Department of Hygiene and Epidemiology, University of Ioannina School of Medicine, Ioannina, Greece; 2 Department of Orthopaedics, University of Ioannina School of Medicine, Ioannina, Greece; 3 Molecular Cell Biology and Regenerative Medicine, Sahlgrenska Academy, University of Gothenburg, Gothenburg, Sweden; 4 Center for Evidence Based Medicine and Department of Biostatistics, Program in Public Health, Brown University, Providence, Rhode Island, United States of America; Memorial Sloan Kettering Cancer Center, United States of America

## Abstract

Systematic reviews that employ network meta-analysis are undertaken and published with increasing frequency while related statistical methodology is evolving. Future statistical developments and evaluation of the existing methodologies could be motivated by the characteristics of the networks of interventions published so far in order to tackle real rather than theoretical problems. Based on the recently formed network meta-analysis literature we aim to provide an insight into the characteristics of networks in healthcare research. We searched PubMed until end of 2012 for meta-analyses that used any form of indirect comparison. We collected data from networks that compared at least four treatments regarding their structural characteristics as well as characteristics of their analysis. We then conducted a descriptive analysis of the various network characteristics. We included 186 networks of which 35 (19%) were star-shaped (treatments were compared to a common comparator but not between themselves). The median number of studies per network was 21 and the median number of treatments compared was 6. The majority (85%) of the non-star shaped networks included at least one multi-arm study. Synthesis of data was primarily done via network meta-analysis fitted within a Bayesian framework (113 (61%) networks). We were unable to identify the exact method used to perform indirect comparison in a sizeable number of networks (18 (9%)). In 32% of the networks the investigators employed appropriate statistical methods to evaluate the consistency assumption; this percentage is larger among recently published articles. Our descriptive analysis provides useful information about the characteristics of networks of interventions published the last 16 years and the methods for their analysis. Although the validity of network meta-analysis results highly depends on some basic assumptions, most authors did not report and evaluate them adequately. Reviewers and editors need to be aware of these assumptions and insist on their reporting and accuracy.

## Introduction

Indirect comparisons between interventions have been frequently conducted in meta-analytic studies during the last few years [1]–[3]. In 1997 Bucher et *al*. introduced the ‘adjusted indirect comparison’ method and established it as a valid statistical tool to infer about the relative effects of two treatments [4]. The method implies that we can indirectly compare treatments B and C (allowing for uncertainty) via a common comparator treatment A using information from ‘A versus B’ and ‘A versus C’ randomized control trials (RCTs). More advanced methods have been developed since and they are used to synthesise direct and indirect evidence over a network of studies that compare many competing interventions. The increasing need to compare more than two alternative treatments and classify them according to their relative effectiveness or safety has underpinned the rapid development of network meta-analysis (NMA).

NMA can be seen under different perspectives. Lumley fitted NMA as a meta-regression model with dummy variables that define the various comparisons [5]. Lu and Ades suggested a hierarchical NMA model fitted in a Bayesian framework by extending the model initially introduced by Higgins and Whitehead [6], [7]. Recently, White et *al.* showed that NMA is a special case of multivariate meta-analysis [8]. The models can be fit in a Bayesian or frequentist software and several approaches to evaluate statistically the assumption of consistency (that is agreement between direct and indirect evidence) have been proposed [9], [10].

The ease of application of the various methods to fit the NMA or to evaluate consistency largely depends on the network structure. For example, data from star-shaped networks (when the treatments in the network have been compared directly to a common reference but not between themselves) can be easily synthesized using any standard meta-regression routine whereas in the presence of multi-arm studies more appropriate (and often more cumbersome) methods are needed. A simple z-test that compares direct and indirect estimates might be enough to evaluate statistically the assumption of consistency in a network with only a couple of closed loops. In contrast, a sophisticated approach like the design-by-treatment interaction model is needed for networks with many loops and multi-arm studies [10]. The prevalence of such important network features (e.g. multi-arm studies, closed loops) can direct methodologists into investing resources in developing statistical models and software that are relevant to the majority of the networks encountered in the medical literature.

The NMA framework has been recently established and consequently the properties of the various methods are still under investigation. The first simulation and empirical studies that evaluate or compare NMA-related methods have recently appeared in the literature [11]–[17]. The simulation studies have been largely designed according to the characteristics of pairwise meta-analyses. However, this might not be appropriate and simulation scenarios should ideally draw on the characteristics of published networks.

In this paper we aim to provide an overview of the characteristics of the published networks of interventions. We anticipate that our results will be a useful resource to investigators planning simulations or empirical studies but will also steer the development of methods towards directions relevant to the majority of the networks rather than special cases. Finally, we aim to explore the uptake of new methodologies by meta-analysts and to investigate whether the choice of a particular NMA methodology is associated with the network’s structural characteristics.

## Methods

### Search Strategy and Eligibility Criteria

We searched PubMed for research articles published until 12/2012 using the following search code: (network OR mixed treatment* OR multiple treatment* OR mixed comparison* OR indirect comparison* OR umbrella OR simultaneous comparison*) AND (meta-analysis). All meta-analyses of RCTs including at least four treatments and any form of indirect comparison were eligible. When the method of indirect inference was not reported, we included the network if the reported indirect estimates were identical or similar to the Bucher method. We excluded meta-analyses of diagnostic test accuracy studies as well as those including observational studies. We also excluded all articles using the naïve approach to derive indirect inferences (e.g. pooling patient outcomes across study arms) [18]. To ensure a substantial mass of data per network we excluded studies in which the number of trials was not greater than the number of competing treatments. We excluded networks with three treatments since the characteristics of such networks have been described in previous studies [3], [12].

### Data Extraction

Four authors (HV, AC, AV, AN) independently extracted data. For all networks published until 12/2012, we extracted the name of first author, year of publication, journal of publication, the primary outcome or (if not specified) the outcome reported first in the analysis, the number of included studies, the synthesis method (when reported), the control intervention (e.g. placebo, no treatment or standard care), the type of outcome, and the number and type of competing treatments. For all networks published up to 3/2011 we also extracted outcome data for the primary outcome or the outcome reported first in the article. We preferred arm-level data, if available, to study-level data.

We categorised the networks that met the inclusion criteria into two categories; star-shaped networks and full networks (networks with one or more closed loops). We categorised each outcome as beneficial or harmful. We categorised each network according to the reported outcome type (objective, semi-objective or subjective) and treatment comparison (pharmacological interventions versus placebo, pharmacological versus pharmacological or non-pharmacological versus any intervention) using previously suggested definitions [19]. Categorisation of outcomes and comparisons is important for making inferences about the amount of heterogeneity expected in the network [19]. If a network included at least one non-pharmacological treatment we categorised it as pertaining to ‘non-pharmacological versus any intervention’ type of comparison. When a network included pharmacological treatments and placebo or control we categorised it to pharmacological versus placebo/control intervention comparison type, whereas when placebo and an obvious control group were absent we categorised it as pharmacological versus pharmacological comparison type. Any disagreements during data extraction were resolved by discussion.

We further categorised networks according to the type of outcome measure into four categories; dichotomous, continuous, time-to-event or rate data. We also recorded the effect size that each network has used in the analysis (for dichotomous data odds ratio (OR), risk ratio (RR), risk difference (RD), for continuous data mean difference (MD), standardized mean difference (SMD) and ratio of means (RoM), and for time-to-event or rates hazard ratio (HR) and rate ratio respectively). Finally we extracted data about the method used to derive indirect inference (Bucher method, meta-regression, Bayesian hierarchical model or multivariate meta-analysis) and the method used to evaluate statistically the presence of inconsistency (such as node-splitting, Lumley model etc.). A description of the methods and their references can be found in Table 1 and Table 2.

**Table 1:** Description of methods to derive indirect and mixed estimates.

Network Meta-Analysis Methods	
Bucher method	Bucher’s method for indirect comparison (also called the adjusted indirect comparison method) is a statistical method to derive an indirect estimate for the relative effectiveness of two treatments via a common comparator. If studies comparing directly the two treatments are also available, their summary effect can be combined with the indirect estimate to obtain the mixed summary effect estimate [4].
Bayesian hierarchical model	This model relates the observed relative treatment effects with their ‘true’ underlying treatment effects in studies that are assumed to be fixed or random around the comparison-specific summary mean effect. Then, the consistency equations link the mean effects. The hierarchical model was first described in [7] for three treatments and extended in [6].
Meta-regression	A meta-regression model with dummy variables that denote the observed direct comparisons that relate to the basic parameters (the smallest set of comparisons that can generate all possible comparisons via the consistency equations). In such a meta-regression model without an intercept the estimated regression coefficients are the network meta-analysis summary treatment effects [3].
Multivariate meta-analysis model	This model treats the different treatment comparisons observed in studies as different outcomes. Using a data augmentation technique to ‘impute’ a common reference arm in all studies, a standard multivariate meta-analysis model can be employed [8]. We included this method for the sake of completeness although we do not anticipate any network to have used it as it was first introduced in 2012.

**Table 2:** Description of statistical methods used to evaluate the consistency assumption.

*Local tests (identify comparisons or loops associated with inconsistency)*	
Loop-specific approach	This method estimates inconsistency as the difference between direct and indirect evidence in each closed loop of the network. The z-test is repeatedly used to assess the assumption of consistency. It is often called the Bucher method [4].
Node-splitting and back-calculation	The node-splitting approach compares the direct and indirect evidence, the latter estimated from the entire network after excluding the comparison of interest.The back-calculation method is based on the same idea but the indirect evidence is calculated as a weighted difference between the NMA and the direct estimate [9].
Caldwell test	A ‘composite’ -test to evaluate inconsistency between the direct and the various indirect estimates derived from all independent loops in the network for each specific comparison [20].
***Global tests (infer about consistency in the entire network)***	
Comparison of model fit and parsimony	A global test using the deviance information criterion (DIC) to infer about the presence of inconsistency in the entire network. Both the standard network meta-analysis model and the inconsistency model (a model equivalent to a series of unrelated pairwise meta-analyses with common heterogeneity) are fit. Then, if the DIC for the inconsistency model is lower by more than three units, the consistency assumption is challenged [21].
Lumley model	A method to estimate inconsistency using a linear model with additional comparison-specific random terms, the common variance of which is a measure of the statistical inconsistency for the entire network [5].
Lu and Ades model	A NMA model that includes an additional term in each loop. These terms (often called inconsistency factors) are usually assumed exchangeable and their common variance is the inconsistency variance in analogy to the heterogeneity variance [22].
Design-by-treatment model	A regression model where additional terms (random or fixed) are used to denote disagreement between study designs, where the latter is defined as the set of treatments compared in a study. This approach is the only one insensitive to parameterization of the multi-arm studies [10]. We included this method in the list for the sake of completeness although we do not anticipate any network to have used it as it was first introduced in 2012.

### Analyses

We derived descriptive statistics for publication characteristics (year of publication, journal) and size-related characteristics such as number of studies and number of treatments per network. We estimated the prevalence of each type of outcome and treatment comparison and the frequency of each statistical method employed for NMA. We describe more in detail networks published up to 3/2011 and we provide network-specific, loop-specific and comparison-specific characteristics as appropriate (such as sample size, number of loops etc.).

Descriptive statistics were calculated separately for star and full networks and jointly when the two categories could be merged. We observed how often different methodologies have been employed over years and we describe the relationship between analysis method and characteristics related to the network size. We present continuous characteristics with the median and interquartile range (IQR) and we compare them in groups using the Mann-Whitney test.

## Results

### Identified Networks

**Figure 1:**
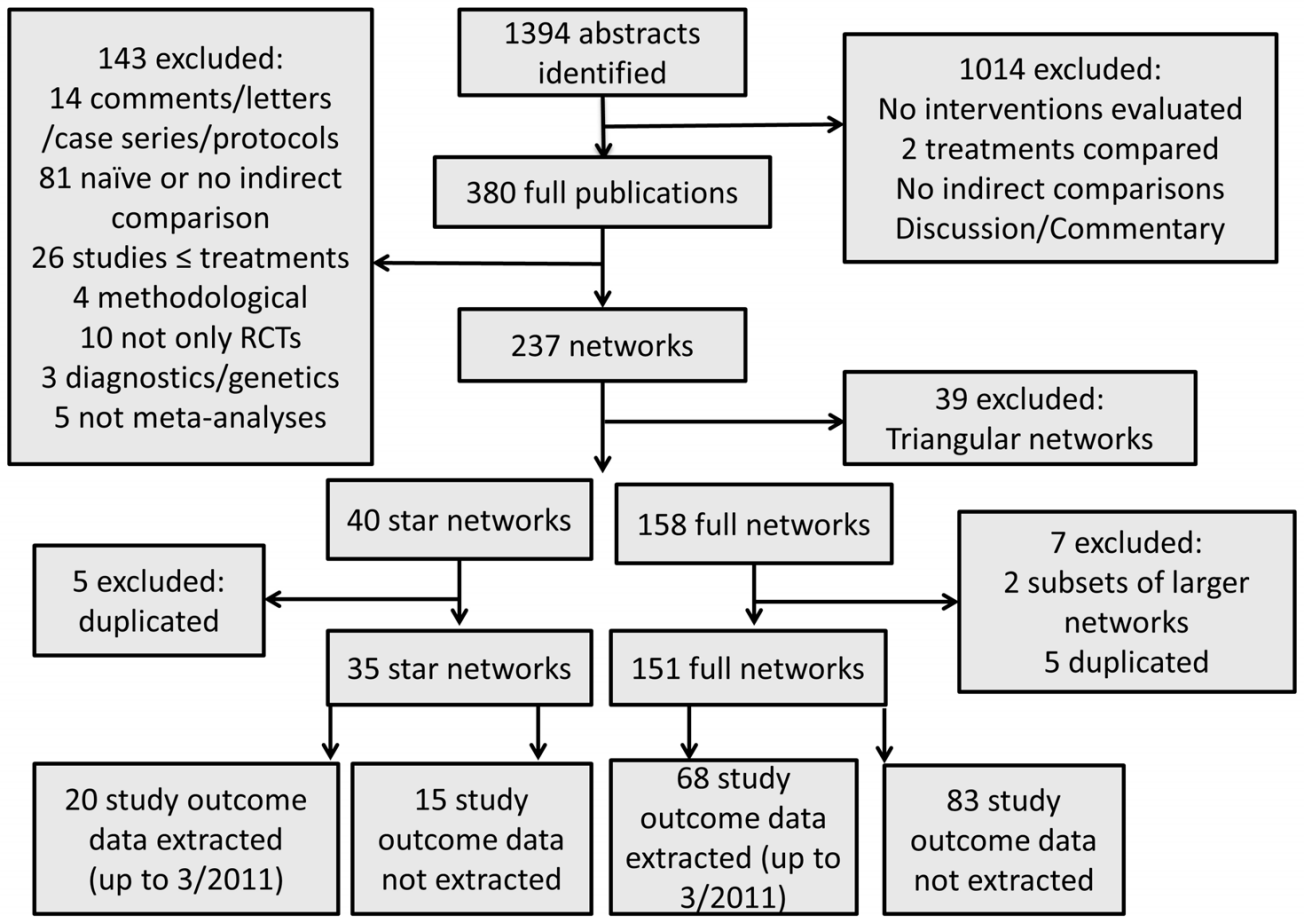
Flow chart of identified networks.

After screening 1394 abstracts, we identified 380 potentially eligible networks of interventions. The full text of these publications was assessed and we ended up with 186 networks that met our inclusion criteria. Out of the total 186 networks, 35 (19%) were star networks and 151 (81%) were full networks. We identified 88 networks published before 3/2011 for which we extracted study outcome data; 20 were star networks and 68 full networks. The network selection process is shown in the flowchart of Figure 1. The full list of the 186 networks and their characteristics can be found in http://www.mtm.uoi.gr/.

### Publication Characteristics

**Figure 2:**
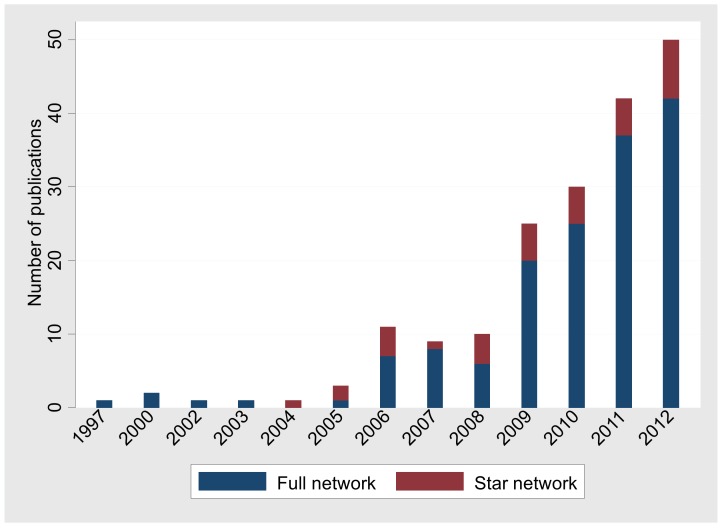
Number of meta-analysis articles with full and star networks published between 1997–2012.

The number of networks published by year is shown in Figure 2. There is a steep increase in the publication of networks with time, which is more pronounced for full networks rather than for star networks.

**Figure 3:**
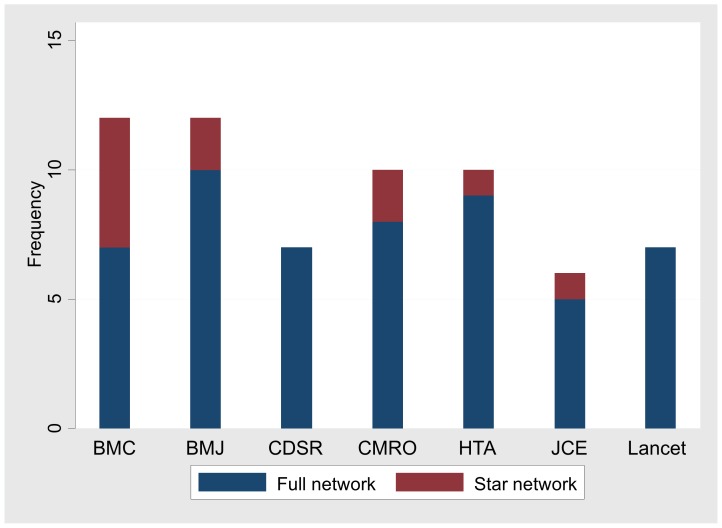
Number of meta-analysis articles with full and star networks published by journal. BMC: BioMed Central BMJ: British Medical Journal CDSR: Cochrane Database of Systematic Reviews CMRO: Current Medical Research & Opinion HTA: Health Technology Assessment JCE: Journal of Clinical Epidemiology.

Most networks were published in British Medical Journal (BMJ) (12 (6%)) and in BioMed Central (BMC) (12 (6%)). Figure 3 shows the number of published networks in the seven most prevalent journals.

### Size and Density Characteristics (Table 3)

Table 3 summarizes the structural characteristics of the networks. In the sample of 186 networks the median number of studies per network was 21 with IQR 13 to 40. The median number of treatments included in a network was 6 (IQR 5 to 9). Full networks appear to contain more studies (median 21) and treatments (median 7) than star networks (median number of studies 19 and median number of treatments 5) (P = 0.096 for the comparison of studies and P = 0.017 for the comparison of treatments). The subset of 88 networks published until 3/2011 had similar characteristics; a median number of studies 22 (IQR 13 to 38) and a median number of treatments 6 (IQR 4 to 9). Full networks published until 3/2011 had a median number of studies 22 and median number of treatments 6 with the respective medians in star networks being 19 and 5 (P = 0.262 for the comparison of studies and P = 0.169 for the comparison of treatments).

**Table 3:** Structural characteristics of full and star networks.

Size and density characteristics	All networks	Full networks	Star networks	Comparison of full and star networks (p-value of Mann-Whitney test)
Median number of studies pernetwork (IQR)	21 (13–40) [186]	21 (13–45) [151]	19 (11–29) [35]	0.096
Median number of treatmentsper network (IQR)	6 (5–9) [186]	7 (5–9) [151]	5 (4–7) [35]	0.017
Median sample size pernetwork (IQR)	7729 (3043–24987) [82]	8491 (4587–27659) [62]	2995 (1829–12499) [20]	0.025
Median sample size percomparison (IQR)	577 (208–1707) [80]	576 (185–1785) [61]	600 (366–1217) [19]	0.181
Median number of studies per comparison (IQR)	2 (1–4) [88]	2 (1–4) [68]	3 (2–6) [20]	<0.001
Median number of loops pernetwork (IQR)	–	4 (1–70) [68]	–	–
Median sample size per loop (IQR)	–	2159 (989–8379) [61]	–	–
Median number of studies per loop	–	8 (6–15) [68]	–	–

Some characteristics could be estimated for all networks (186, published until 12/2012) whereas some other characteristics require outcome data and were estimated from 88 networks published until 3/2011 or their subsets. The exact number of networks evaluated in each case is given in square brackets. In parenthesis we present the interquartile range.

Out of the 88 networks published until 3/2011, for 6 full networks that reported study-level outcome data (that is effect sizes and variances) we could not estimate the sample size in the network and for 8 (7 full networks and 1 star network) we could not estimate the sample size per comparison. The overall median sample size per network (estimated in the remaining 82 networks) was 7729 with IQR 3043 to 24987. The median sample size per full network (8491 patients) was considerably larger than median sample size per star network (2995 patients, P = 0.025). However, the median sample size per comparison in full networks was 576 (IQR 185 to 1785), whereas in star networks it was slightly larger (median 600, IQR 366 to 1217, P = 0.181). Star networks tend to have also a larger number of studies per comparison (median 3) than full networks (median 2, P<0.01). Thus, full networks are larger than star networks in terms of total number of studies, treatments and sample size but star networks are more ‘dense’ having larger number of studies and patients per comparison. Star networks could be described as more compact networks; examine fewer comparisons than full networks but these comparisons contain more data.

### Characteristics of the Primary Outcome (Table 4)

The primary outcome was an objective outcome in 36 (19%) out of 186 networks, 72 (39%) networks had a semi-objective primary outcome and 78 (42%) a subjective outcome. In almost half of the 186 networks (91, (49%)) the primary outcome was beneficial. The majority (111 (60%) networks) had a dichotomous primary outcome and 53 networks (28%) had a continuous outcome. Less often networks had time-to-event (17 (9%) networks) or rate (5 (3%) networks) primary outcomes. Out of 111 networks with a dichotomous outcome 66 (59%) employed OR, 44 (40%) RR, none used RD and one (1%) used all three effect sizes (OR, RR and RD). Out of 53 networks that used a continuous outcome 43 (81%) reported results on MD scale, 9 (17%) used the SMD and one used RoM. All 17 networks with time-to-event data employed HR and the 5 networks with rate data employed rate ratio. Star networks had a dichotomous outcome more often than full networks (77% vs 56%). Out of 88 networks published by 3/2011, one in four (20 networks) reported study-level data (relative treatment effects and variances) whereas three quarters (68 networks) reported arm-level data.

Table 4 summarizes the outcome characteristics of the 186 full and star networks.

**Table 4:** Characteristics of the primary outcomes and their measures in full and star networks published until 12/2012.

	Full networks 151	Star networks35	Total186
**Type of outcome**			
Objective	29 (19%)	7 (20%)	36 (19%)
Semi-objective	66 (44%)	6 (17%)	72 (39%)
Subjective	56 (37%)	22 (63%)	78 (42%)
**Outcome measured as**			
Dichotomous	84 (56%)	27 (77%)	111 (60%)
Continuous	47 (31%)	6 (17%)	53 (28%)
Time-to-event	15 (10%)	2 (6%)	17 (9%)
Rate	5 (3%)	–	5 (3%)
**Effect size**			
OR	57 (37%)	9 (26%)	66 (35%)
RR	26 (17%)	18 (51%)	44 (23%)
OR RR RD	1 (1%)	–	1 (1%)
HR	15 (10%)	2 (6%)	17 (9%)
Rate ratio	5 (3%)	–	5 (3%)
MD	39 (26%)	4 (11%)	43 (23%)
SMD	7 (5%)	2 (6%)	9 (5%)
Ratio of Means	1 (1%)	–	1 (1%)

The table shows the number of networks and the respective percentage in parenthesis.

### Treatments Compared in Networks (Table 5)

The 186 networks evaluated a wide range of interventions (Table 5). The most common comparison type was pharmacological intervention versus placebo or control (129 networks, 69%). In 36 (19%) networks the comparison type was non-pharmacological versus any intervention and 21 (12%) networks compared only pharmacological interventions. Six networks (3%) included both placebo and control, 117 networks included only placebo (66%) and 26 networks included control or no treatment but not placebo (15%).

**Table 5:** Characteristics of the treatment comparisons in full and star networks published until 12/2012.

	Full networks 151	Star networks 35	Total 186	
**Intervention comparison type**				
Pharmacological vs pharmacological	16 (11%)	5 (14%)		21 (12%)
Pharmacological vs placebo/control	99 (65%)	30 (86%)		129 (69%)
Non- pharmacological vs any	36 (24%)	–		36 (19%)

The table shows the number of networks and the respective percentage in parenthesis.

### Network Meta-Analysis Methods (Table 6)

In our sample of 186 networks, the most frequent method employed to synthesise the data was the Bayesian hierarchical model reported in 113 (61%) networks (Table 6). Meta-regression (28 (15%) networks) and Bucher method of indirect comparison (29 (15%) networks) were also widely used in the published networks.

**Table 6:** Methods employed to synthesise data in full and star networks published until 12/2012.

Network Meta-Analysis method	Full networks 151	Star networks 35	Total 186
Bucher method	17 (11%)	11 (31%)	28 (15%)
Bayesian hierarchical model	98 (65%)	13 (37%)	111 (59%)
Meta-regression	25 (16%)	2 (6%)	27 (15%)
Bucher method and Bayesian hierarchical model	1 (1%)	–	1 (1%)
Meta-regression and Bayesian hierarchical model	1 (1%)	–	1 (1%)
Not reported	9 (6%)	9 (26%)	18 (9%)

For a description of the network meta-analysis methods see Table 1. The table shows the number of networks and the respective percentage in parenthesis.

Methods for indirect comparison varied between full and star-shaped networks. Most full networks used Bayesian hierarchical models (100 (65%)) and one in ten networks (18 (11%)) used the Bucher method for indirect comparisons. Only 13 (37%) star networks employed a Bayesian hierarchical model and 11 (31%) used the Bucher method. The proportion of networks performing meta-regression was greater in full than star networks (17% vs 6%). Finally, over one in four star networks (9 (26%)) did not report which synthesis method they used whereas the respective proportion in full networks was only 6% (9 networks).

**Figure 4:**
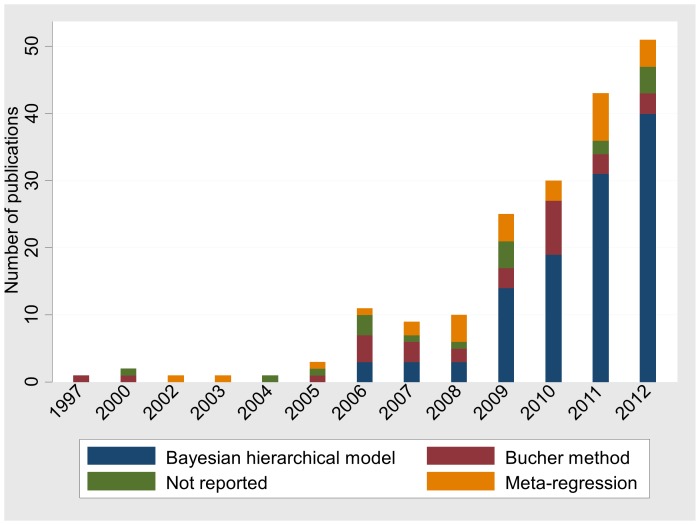
Number of published networks by year (1997–2012) and the Network Meta-Analysis method. Networks that used more than one method are included in all relevant categories.

The methods used to synthesise evidence seem to have changed over time. Figure 4 shows the number of networks published between 1997 and 2012 according to the synthesis method. In the networks published before 2008 (39 networks) Bucher was the most prevalent method (12 (31%) networks), followed by meta-regression (10 (26%) networks) and Bayesian hierarchical model (9 (23%) networks). Over 71% of the 147 networks published after 2009 used a Bayesian hierarchical model (104 networks) while the Bucher method and meta-regression were less frequently employed. What is alarming, however, is that a sizeable number of articles did not specify the analysis method and this number has not changed much during the last six years (11% of networks published in 2007, 5% in 2011 and 8% in 2012).

Networks analyzed with a Bayesian hierarchical model had a median number of studies 21 (IQR 14 to 45) and a median number of treatments 7 (IQR 5 to 9). The size of networks that used the Bucher method was smaller having a median number of studies 19 (IQR 11 to 38, P = 0.569) and median number of treatments 5 (IQR 4 to 8, P = 0.014). Networks using meta-regression had a median number of studies 20 (IQR 13 to 31, P = 0.423 compared with the Bayesian hierarchical model) and median number of treatments 7 (IQR 5 to 8, P = 0.174 compared with the Bayesian hierarchical model). The size of the network did not differ between networks that employed meta-regression and those that employed the Bucher method neither in terms of number of studies (P = 0.848) nor in terms of number of treatments (P = 0.259). Most recently published networks (after 2009) used a Bayesian hierarchical model whereas the most prevalent method before 2009 was the Bucher method. The popularity of the hierarchical model in the last years cannot be fully attributed to the fact that recently published networks are larger and dense. The median number of studies and the median number of treatments do not seem to differ much between networks published before 2009 (median number of studies 19 (IQR 14 to 38) and median number of treatments 6 (IQR 4 to 8)) and after 2009 (median number of studies 21 (IQR 13 to 40) and median number of treatments 7 (IQR 5 to 9)) (P = 0.872 for the comparison of studies, P = 0.150 for the comparison of treatments).

### Characteristics of Closed Loops of Evidence and Evaluation of Inconsistency (Table 7)

To examine the prevalence of closed loops in networks, we consider the 68 full networks for which we had outcome data (Table 7). We found that the majority included at least one three-arm trial (56 (82%) networks) and 18 networks (26%) included at least one four-arm trial. The median number of two-arm trials per network was 19 (IQR 11 to 31) and the median number of three-arm trials per network was 2 (IQR 1 to 4). The number of loops per network had IQR 2 to 9 with median 4 and the total number of loops from the 68 networks was 426.

**Table 7:** Statistical methods used to evaluate consistency in 151 full networks published until 12/2012.

Method employed	Full networks 151
**Appropriate statistical methods**	
Loop-specific approach	22 (14%)
Lumley model	10 (7%)
Lu and Ades model	1 (1%)
Node-splitting	9 (5%)
Comparison of model fit and parsimony	2 (2%)
Combination of appropriate statistical methods	4 (3%)
**Inappropriate methods**	
Comparison of network estimates with the direct estimates	21 (14%)
Informal comparison of the results with previously conducted meta-analyses	14 (9%)
Informal comparison of indirect estimates with the direct estimates	1 (1%)
**None reported**	
None reported	67 (44%)

For a description of the methods to evaluate inconsistency see Table 2. The table shows the number of networks and the respective percentage in parenthesis.

Out of the 151 identified full networks, the assumption of consistency was evaluated by using the loop-specific approach in 22 (14%) networks. Ten (7%) networks used the Lumley model to evaluate inconsistency, whereas 9 (5%) performed the node-splitting method. The Lu and Ades model was employed to evaluate consistency in one network; in 2 networks (2%) the authors performed comparison of model fit and parsimony. Four (3%) networks used combinations of appropriate statistical methods to evaluate inconsistency such as the loop-specific approach and comparison of model fit and parsimony (2 networks), Lu and Ades model and comparison of model fit and parsimony (1 network). In 36 networks (24%) the authors used inappropriate methods to evaluate inconsistency. A popular but inappropriate method was the comparison of direct and estimates derived from NMA which was performed in 21 (14%) networks; this approach is inappropriate because the network estimate comprises the direct estimate and hence they are not expected to differ much. In 14 (9%) networks the authors compared informally (without using an appropriate statistical tool) their results with results from previous meta-analyses and in one network the authors compared informally direct to indirect estimates (see Table 7).

**Figure 5:**
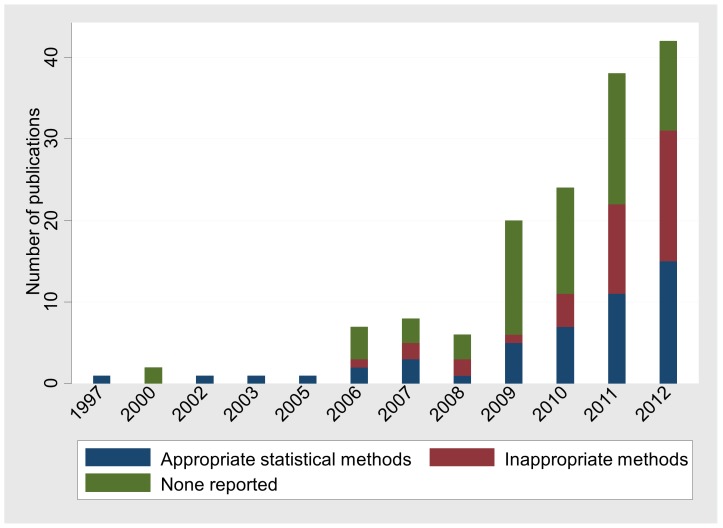
Number of published full networks by year (1997–2012) and the method employed to examine inconsistency. Appropriate statistical methods are presented in Table 2. Networks that used more than one method are included in all relevant categories.

Authors’ awareness about the importance of evaluating the consistency assumption has increased during the last few years and they employ statistical methods more frequently than before (Figure 5). Fewer than half (42%) of the networks published in 2011 did not report or did not evaluate the assumption of consistency whereas the respective proportion in networks published in 2012 was 26%.

## Discussion

NMA is increasingly used in medical literature and provides a useful contribution to evidence based decision making. The ability to compare treatments that have never been compared directly, the increase in power and precision and the potential of NMA to provide a ranking of the available treatments are the main advantages of the methodology.

Previous studies have explored the characteristics of networks of interventions using indirect comparisons to evaluate different aspects of the NMA methodology [11], [14], [23]. The recently published article by Bafeta et *al.* employed slightly different eligibility criteria to end up with 121 networks published until 7/2012 [24]. The results of their study are comparable with ours for those characteristics evaluated in both papers (e.g. median number of treatments and studies). Our study was however more focused on the statistical aspects of the methodology whereas Bafeta et *al.* yielded more information about the general review methodology employed; hence the two studies can be thought of as complementary. For instance Bafeta et *al.* reported that half of the networks (44%) did not mention the consistency assumption and we found that only one in three (32%) networks undertook appropriate statistical methods to evaluate inconsistency. In our study we placed more importance on structural characteristics that are associated with important methodological aspects (such as the presence of multi-arm studies and closed loops) and we extracted outcome data to provide more information about sample size. On the other hand, Bafeta et *al.* investigated and found that the reporting of the search strategy, the assessment of risk of bias and the evaluation of publication bias was suboptimal in many network articles.

Our results show that there is substantial variation in the statistical methodological approaches used to synthesize evidence across networks. Until recently, it was easier to account for correlations induced by multi-arm studies and to estimate the probabilities for each treatment of being the best within a Bayesian framework. The flexibility of this specific approach possibly explains why most investigators choose a Bayesian hierarchical model to synthesize evidence (61%). This finding is in line with other studies that conclude that Bayesian hierarchical models have been increasingly used [11], [25], [26]. An inconsistent network of interventions is unlikely to form a reliable basis for choosing the best available intervention for a specific condition. Despite that, many NMA publications did not employ or did not report the use of any method to evaluate inconsistency (44%) or they used informal and inappropriate methods to do so (24%).

Evaluation of inconsistency and model fitting become more complex in the presence of multi-arm studies as within-study they are consistent by definition [27]. We found that full networks include a median number of 2 multi-arm studies and that the presence of multi-arm studies is likely. Consequently, investigators and trainers should use methods that are more complex but account for the implications of multi-arm studies in the data, such as the design-by-treatment model [10].

One limitation of our study is that we may not have included all published meta-analyses that performed indirect comparisons because some may not have been indexed using the search code specified. Furthermore, networks of interventions could be identified only if they were indexed in PubMed. However, we think that our database is a representative sample of published networks of interventions in medical literature. This is also supported by the fact that our results are comparable to those reported by Lee who conducted a review of network meta-analyses up to 6/2012, searched more databases as well as conference abstracts [28]. Our reliance on the information reported by authors about the methodologies employed might also have impact on our study’s conclusions. Authors may have used appropriate statistical methods to synthesize evidence and evaluate inconsistency but have reported them inadequately. It has been shown that reporting of NMA is suboptimal [11], [23], [24] and guidelines based on consensus are needed. Despite these limitations, to our knowledge this is the largest study exploring and describing in detail the structural and analytical characteristics of networks of interventions.

### Conclusions

Our descriptive analysis offers an insight into the characteristics of networks of interventions over the last 16 years. The typical network included in our database is a network with a dichotomous semi-objective outcome and compares pharmacological interventions vs placebo. It includes 6 treatments examined in 21 studies. It is likely to be a full network with 3 closed loops of evidence, 2 three-arm and none four-arm trial. A Bayesian hierarchical model is the most popular method to synthesise the data. However, the use of appropriate methods to evaluate the assumptions underlying NMA is still limited, moderating the strength of studies’ conclusions. Awareness of assumptions by authors, reviewers and editors is crucial to improve reporting of relevant methodological aspects.
